# An Integrated Study on the Fading Mechanism of Malachite Green Industrial Dye for the Marquisette Curtain in the Studio of Cleansing Fragrance, the Palace Museum (Beijing)

**DOI:** 10.3390/molecules27144411

**Published:** 2022-07-09

**Authors:** Le Wei, An Gu, Zhimou Guo, Junjie Ding, Gaowa Jin, Yong Lei

**Affiliations:** 1 Conservation Department, The Palace Museum, Beijing 100009, China; gu.an@outlook.com; 2 CAS Key Laboratory of Separation Sciences for Analytical Chemistry, Dalian Institute of Chemical Physics, Chinese Academy of Sciences, Dalian 116023, China; guozhimou@dicp.ac.cn; 3 Ganjiang Chinese Medicine Innovation Center, Nanchang 330000, China; dingjunjie@dicp.ac.cn

**Keywords:** marquisette curtain, fading mechanism, malachite green, degradation pathways, metabolomics

## Abstract

Historical marquisette curtains were composed of lightweight fabrics, woven in an open-mesh and leno-type weave, usually made of silk, and found in Qing imperial buildings. As panel curtains, they were exposed to light, and so underwent fading. This study investigated the manufacturing technology and fading mechanism of dyed marquisette fabric from the Studio of Cleansing Fragrance, the Palace Museum (Beijing). The technological aspects were identified. The types of weave, fiber, and adhesive used to fix the curtain to the wooden frame were identified through microscopic observation and infrared spectroscopy. A color change characterization was performed based on UV-visible diffuse reflectance spectra. The textile colorant was identified as malachite green (MG), and its degradation by light was subsequently studied by dynamic photolysis experiments in a kinetic solution for the rapid exploration of by-products. The main degradation pathways were thus identified and the factors responsible for the induced color changes were discussed. A comparison of the liquid chromatography-mass spectrometry (LC–MS) results of the products derived from the photolysis method as well as of the samples extracted from the object allowed for the identification of the presence of different degradation pathways in the faded and unfaded parts of the textile. A metabolomics analysis was applied to account for the differences in the degradation pathways.

## 1. Introduction

Historic textiles provide valuable information about both the technological history and culture of their manufacturers, but the materials and processes used in their production often predestine them to rapid deterioration. Environmental factors are often the culprit, and the influence of light is one of the most detrimental degradation agents, leading to structural damage and color change in textiles, especially in collections illuminated with natural and display lighting [1]. Understanding photodegradation mechanisms to better tailor conservation and preservation measures is crucial for decorative historical interior objects.

Dyestuff research is an important contribution to the understanding of textile objects and a tool in the analysis of the history of textile manufacturing. Inorganic minerals and natural dyes, including plant and insect dyes, were used in textile dyeing from prehistory to the late 19th century [2]. The advent of coal tar dye—aniline purple, synthesized by William Perkin in 1856—set forward the development of synthetic dyes, which progressively replaced natural dyes due to its availability, efficacy and low costs of production [3].

Malachite green (MG), blue-greenish in color but different from the inorganic mineral green pigment malachite, was first synthesized by O. Fischer from benzaldehyde in 1877, then improved by Doebner with benzotrichloride in 1878 taking the generic name of Basic Green 4 [4], and mainly applied in soft furnishings and washing fabrics for a jade green hue until 1920 [5], in addition to seeing non-textile usage, such as in paper, lacquers, and ink dyeing [6]. There are extensively referenced data on the range of the use of synthetic dyes of this triphenylmethane type in textile conservation. Tamburini et al. explored MG in the production of Central Asian *ikat* textiles in the nineteenth century [7]. Liu et al. reported it as an early synthetic dye application in the textile World Exposition in the late nineteenth century in China [8]. Although it has excellent qualities, such as brilliant shades and high tinting strengths, the color could change greatly, resulting from the chemical processes that occur on its exposure to light [9]. The degradation of MG has been studied mainly in the context of industrial dye pollution. The photooxidative degradation of MG has been investigated by the means of an advanced oxidation process using ultraviolet radiation with H_2_O_2_ as an oxidation agent, and a kinetic model was proposed that involved a radical mechanism via two competitive processes of N-demethylation and the destruction of the conjugated structure [10]. A study on the biodegradation of MG dye by *Trichoderma asperellum* laccase confirmed the transformation of benzaldehyde metabolites via a Michler’s ketone pathway [11].

Reversed-phase liquid chromatography is currently the most suitable analytical strategy routinely employed in the analysis of organic natural and synthetic dyes, due to the presence of polar hydrophilic functional groups in these compounds [12]. The identity of dyes can be inferred from their characteristic UV-Vis absorption spectra, collected with photodiode array (PDA) detectors. If coupled with mass spectrometers, the identity of the compounds can be further confirmed based on the patterns of molecular fragmentation [13]. However, when testing historic objects whose colors have faded or otherwise changed, the results of liquid chromatography–mass spectrometry (LC–MS) analyses are difficult to interpret, because the original dyes oftentimes undergo decomposition or conversion. Herein, metabolomics as a comprehensive platform, based on LC–MS, was applied to improve the results’ accuracy [14]. Additionally, a chemometric approach was used to compare data and assess the characterization and authentication of dyes [15]. This multivariate analysis allows highlighting the differences and similarities between samples in addition to the correlation between variables by means of methods such as unsupervised principal component analysis (PCA) or supervised orthogonal partial least squares discriminant analysis (OPLS-DA). Dye profiles have been evaluated with the help of similar methodologies. Núñez et al. accomplished the rapid screening and discrimination of the phytochemicals of turmeric dyes by evaluating polyphenolic and curcuminoid profiles using liquid chromatography coupled with high-resolution mass spectrometry (LC-HRMS) [16]. Serrano et al. discriminated between American cochineal species of 72 historical specimens dating from the 19th century via high-performance liquid chromatography with a diode array detector (HPLC-DAD) accompanied with chemometric methods [17]. Berbers et al. assessed the chemical composition of lac insect dyes lac via high-performance liquid chromatography coupled with a diode array detector followed by electrospray ionization and quadrupole time-of-flight detection (HPLC-DAD-ESI-Q-ToF), and established correlations between chemical compositions and production methods via PCA [18].

## 2. Materials and Methods

### 2.1. Analyzed Object Description

Leno fabrics that are lightweight and smooth in texture have been typically applied in summer clothing, embroideries, and ornaments, such as marquisette curtains, in an open-mesh weave since antiquity [19]. Precious leno jacquard, from the Qin and Han Dynasties of imperial China, is one typical representative of ancient Chinese silk fabrics with various patterns woven on its background. The auspicious and longevity patterns could be formed by beating up the wefts with reeds during the weaving. The art of weaving leno gradually faded during the Ming and Qing Dynasties (1368–1911) and was replaced by more complicated craftsmanship such as satin and kesi.

**Figure 1:**
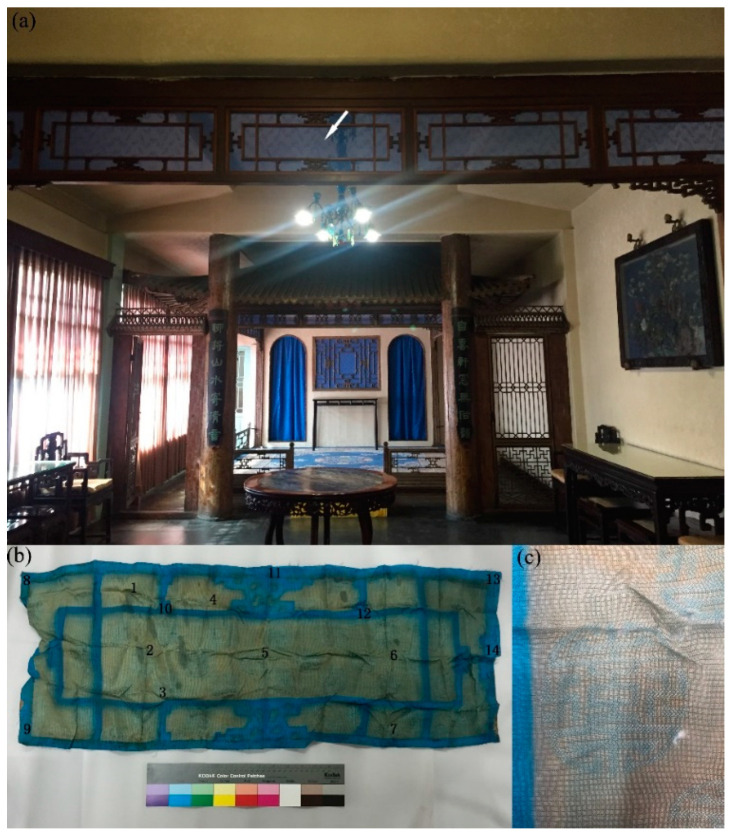
(**a**) Marquisette curtain in the Studio of Cleansing Fragrance (arrow points to its original location); (**b**) the original appearance, including a micro-analysis marked with numbers 1–14; and (**c**) the amplified traditional auspicious and longevity decorated pattern.

The Studio of Cleansing Fragrance was constructed in 1420 as a maid’s residence during the Ming Dynasty (1420–1644), as a prince’s residence during the early Qing Dynasty (1644–1736), and underwent extensive restoration by the Qianlong Emperor (1736–1795). However, according to the synthesis time of malachite green, the marquisette curtains dyed by MG were applied as panel curtains at least after 1878. The curtains were decorated as historical interiors and illuminated with sunlight; they were detached in 2015 for the reparation of the building, and thus their service time was more than 100 years. The Studio of Cleansing Fragrance is an H-shaped hall and consists of front and rear courtyards that are connected with corridors. The smaller stage located in the western bay of the rear hall is used as a tea banquet for the emperor and civil officials on a chosen auspicious day during the Qianlong Emperor’s reign. The photograph of the curtain (95 cm × 36 cm, Figure 1a) showed its original location, which was in the north and south positions of the smaller stage, and it was removed for hanging new ones for visits and receptions. Yu always found this kind of marquisette curtain, which was made of a single layer with a common structure, in ancient Chinese buildings of “wooden frame-curtain-wooden frame” in the Hall of Mental Cultivation [20]. Due to the long-term effects of sunlight, most of the parts that were exposed to the air had obviously faded, and little parts blocked by the wooden window still kept their original lake blue color (Figure 1b). A circle tuanshou pattern filled with two symmetric swastikas as a characteristic unit was woven over the whole fabric (Figure 1c). As a traditional Chinese ornament meaning auspiciousness and longevity, it is commonly found on brocades, hollow doors, and windows in the Ming and Qing Dynasties. 

### 2.2. Chemicals and Reagents

Commercial standard MG (a monochloride salt of bis(4-dimethylaminophenyl)phenylmethylium) dye was purchased from Sinopharm Chemical Reagent Co., Ltd. (Shanghai, China) and used as received without further purification. Dimethyl sulfoxide (DMSO, HPLC grade), formic acid (analytically pure) and hydrochloric acid (37%) were obtained from Fisher Scientific (Waltham, MA, USA). The solvents methanol (MeOH) and acetonitrile were acquired from Merck (Darmstadt, Germany, MS grade). Millipore Elix Advantage 15 ultrapure water system (Molsheim, France) was used throughout the whole experiment with a resistivity of 1.8 × 10^7^ Ω⋅cm. Leucine Enkephalin and sodium formate were purchased from BOC Sciences (New York, NY, USA, MS grade).

### 2.3. Microscopic Observation

The identification of the fiber and weaving pattern was performed with a light microscope (a low-power Leica MZ 16A and a high-power Leica DVM5000, respectively). A Y172 (Shanghai Precision Instruments Co., Ltd., Shanghai, China) Hastelloy slicer was used to observe the cross-section characteristics of the fiber. A morphology analysis was carried out by a high-vacuum Jeol JSM-6360LV scanning electron microscope (SEM) at an acceleration voltage of 8 kV and a working distance of 24 mm. The sample was gold-coated. UV imaging for the curtain was undertaken with a Canon camera at maximum exposure only under UV radiation in a darkroom.

### 2.4. FTIR Spectroscopy

FTIR spectroscopy was conducted in order to characterize the fiber and adhesive types. Infrared spectra were obtained with a Thermo Fisher, Nicolet iN10 Mx infrared spectrometer with a mercury cadmium telluride (MCT) detector cooled with liquid nitrogen. The measurement mode adopted micro transmission with a diamond compression cell. The spectrum was composed of 64 scans and ranged from 4000 to 650 cm^−1^. The spectral resolution was about 4 cm^−1^, and the spectrum was analyzed with OMNIC Picta software.

### 2.5. Color Measurements

Color measurements and UV–visible diffuse reflectance spectra were performed with a Konica Minolta CM-26d model spectrophotometer under the standard condition of D65 and 10° observer, and were analyzed with Spectra Magic NX software. The spectra were in the 360–740 nm range. The Specular Component Excluded (SCE) mode was used for measuring the matt surface of textiles. The recorded CIELAB L*, a* and b* values are presented in Table S1 in the Supplementary Materials.

### 2.6. Solvent Kinetic Accelerated Photoageing Conditions

An artificial accelerated photoageing test was conducted using an Atlas Suntest XLS+ chamber fitted with xenon lamp as a light source working at an irradiance of 550 W/m^2^. A simulation of direct sunlight without a filter was set up, ranging from 300 to 800 nm. The total radiant exposure was calculated as 201,960 KJ/m^2^, with a total exposure time of 102 h.

### 2.7. Sample Production and Aging Procedure

The standard solution of reference MG was prepared by dissolving 3.1 mg of powder into 1.5 mL of methanol. The reagent usage of methanol could be more justified by its compatibility and antimicrobial properties in the entire liquid chromatography system. A kinetic solution irradiated under solar light was conducted and 31 sets of aging samples (10 μL of standard solution into 1 mL of methanol each time) were sampled at different aging time. The reference samples were marked with numbers of K0–K30, respectively, and the corresponding irradiation doses with different aging degrees were recorded in Table S2 in the Supplementary Materials. The characteristic optical images of visible color changes under different aging time of K0, 0 h; K26, 72 h; K27, 78.5 h; and K28, 96 h were displayed in Figure S1.

### 2.8. Sample Preparation

The extraction was carried out first to confirm the specific coloring substance. Textile samples were extracted with 0.2 mL of DMSO for ultrasonic extraction at 75 °C for 10 min. The supernatant was aspirated with a syringe and centrifuged at 10,000 rpm for 3 min. The leftover residues were heated with 0.2 mL of a HCl (37%): MeOH:H_2_O = 2:1:1 (volume ratio) solution at 100 °C for 10 min and filtered out before drying up with nitrogen. The concentrated sample was redissolved with a DMSO solvent containing the first extract and transferred into a clean insert for an injection volume of 2 μL to be tested.

### 2.9. Characterization of Colorant and Degradation Products

Ultra-high-performance liquid chromatography coupled with quadrupole/time-of-flight mass spectrometry (UPLC-QTOF MS) was equipped with two detection sets of chromatographic separation and mass spectrometry systems, including Waters ACQUITY H-Class UPLC with a PDA detector and Waters Xevo G2-XS Q-TOF MS with an electrospray ionization (ESI) ion source. The analytical conditions were as follows: The separations were carried out on ethylene bridged hybrid (BEH) C18 column (2.1 × 100 mm, 1.7 μm); the temperature was set at 30 °C. The UV detective wavelength ranged from 190 to 800 nm. The flow rate was 0.3 mL/min. The mobile phase was composed of water containing 0.1% formic acid as solvent A and acetonitrile as solvent B. The gradient was 95% A from 0 to 0.3 min, 95–5% A from 0.3 to 9.3 min, 5% A held for 1 min, then 5–95% A from 10.3 to 10.5 min and held at 95% A for 1.5 min. Instrument control and data acquisition were performed using MassLynx V4.1 software. Data processing was conducted using the UNIFI 1.9.3, MS^E^ technique with all of the data all the time and the MSMS method in sensitivity mode. The scan range was from 50 to 1200 *m/z*. Other conditions were optimized as follows: source temperature, 120 °C; cone voltage, 40 V; capillary voltage, 3 kV; desolvation gas (N2) flow rate, 600 L/h; nebulize gas flow rate, 10 L/h; desolvation temperature, 450 °C; and collision energy, ramp high energy from 20 to 45 eV. Real-time calibration (every 30 s) applied leucine enkephalin (0.4 ng/μL) as an internal standard.

### 2.10. Multivariate Analysis

Fourteen samples were chosen from different positions on the curtain, and the extraction protocol followed the sample pretreatment conditions. A series of quality controls (QCs) was used by mixing all of the sample extracts to verify the reproducibility and accuracy of the data. Ten needles of QC solutions were injected before the relic extracts to balance the whole system, i.e., QC1–10, and then injected once every seven sample sequences for QC11 and QC12, respectively. PCA and OPLS-DA were analyzed with Waters Nonlinear Dynamics Progenesis QI 3.0.3 and EZinfo 3.0. The identification and clarification used the elucidation tools and 271 online data sources from ChemSpider and offline databases that included basic lipids and fatty acids in their search configuration platforms.

## 3. Results and Discussion

### 3.1. Weaving Structure and Fiber Examination

**Figure 2:**
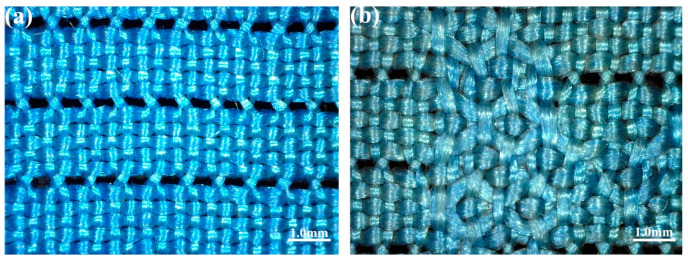
The observation of the plain weave structure of the marquisette curtain (**a**) ×50; (**b**) ×50.

**Figure 3:**
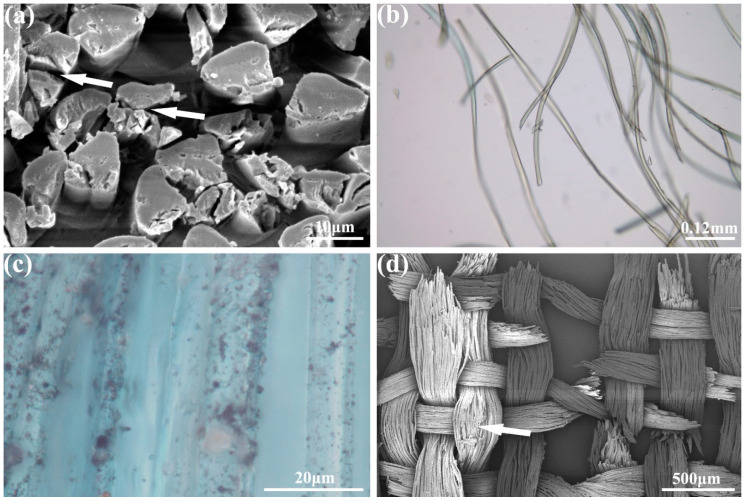
The fiber microphotographs under the SEM (**a**) ×2000; (**d**) ×50 and stereomicroscope (**b**) ×400; (**c**) ×4200, respectively (arrows show the damage, cracks, and fractures of the silk).

The microexamination of some structural features of the curtain showed a typical monochrome plain weave. There were no differences in color between the wefts and warps. The fabric was woven in a leno weave, i.e., after weaving every five weft yarns, the warp yarns were twisted once to form a horizontal strip of plain weave and twisted holes on the surface of the fabric (Figure 2a,b), and this process was called five weft horizontal leno. The observation under the SEM showed that the cross-section was triangular in shape as a whole (Figure 3a). The longitudinal surface displayed a smooth cylindrical shape and the prismatic shape of the silk could be viewed at some angles (Figure 3b), so mulberry silk could be determined. The deterioration degree of the textile fibers is exhibited in Figure 3c. The SEM images showed serious damage, including a decrease in the mechanical strength of the fabric (Figure 3d) and cracks and fractures in the cross-section (Figure 3a).

### 3.2. FTIR Analysis

**Figure 4:**
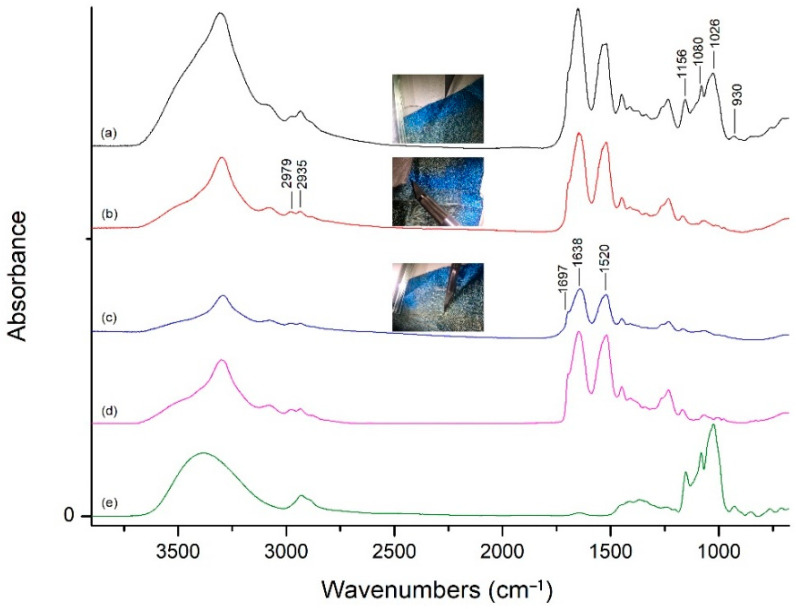
FTIR spectra of the curtain fabric: (**a**) unfaded outer border with adhesive; (**b**) unfaded inner edge without adhesive; (**c**) faded textile; and standard spectra of native mulberry silk (**d**) and starch (**e**).

The absorbance peaks typical for mulberry silk were depicted in Figure 4. A N-H/O-H broad stretching peak was around the region of 3500–3000 cm^−1^; C-H stretching bands of CH_2_ groups were at about 2979 and 2935 cm^−1^. The absorbance bands of amides C=O stretching and C-N-H bending absorbances were centered at 1638 and 1520 cm^−1^, respectively [21]; the former belonged to the Amide I band because of the overexpression of intermolecular β-sheets, while the aged silk additionally exhibited a shoulder band at 1697 cm^−1^, which contributed to its underexpression [22], and the latter was assigned to the Amide II band. The adhesive that was used to fix the curtain to the wooden frame in the Studio of Cleansing Fragrance was detected in the outer border of the unfaded lake blue area, and its characteristic peaks between 1150 and 950 cm^−1^ in the fingerprint region belonged to C-O-C ether bond vibrations of the polysaccharides that were attributed to starch amylose chains [23]. The skeletal vibration of α-1,4 glycosidic linkages in starches was at the absorption band of 930 cm^−1^ [24]. As a result, the presence of starch was confirmed in the unfaded border.

### 3.3. LC-MS Identification

**Figure 5:**
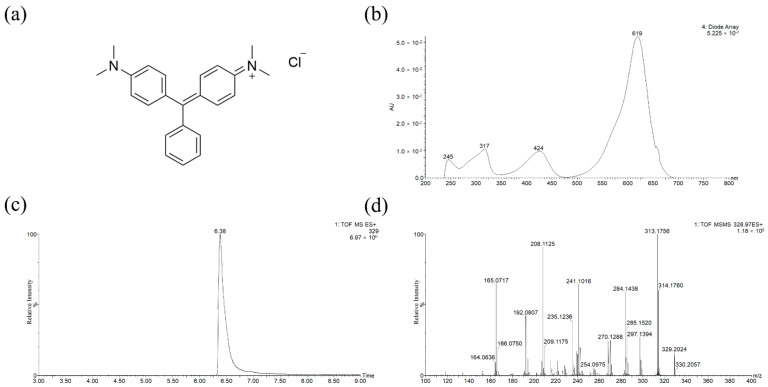
MG chromatogram and spectra: (**a**) molecular structure; (**b**) UV–visible absorption spectrum; (**c**) extracted ion chromatogram (EIC); and (**d**) MSMS spectrum with pseudo-molecular ion *m/z* 329 in a positive ion mode.

Interestingly, the borders exposed fluorescence phenomenon under UV light (Figure S2). Indigo was the most commonly used blue dye in court dyeing but did not fluorescence under UV-induced illumination [25]. Additionally, due to its dyeing properties, it was difficult to dye green hues. To explore the fading mechanism, it is necessary to identify the dyes on textiles first. The presence of MG was confirmed according to the UV-Vis (absorbance peaks at λ_max_ 619, 424, 317 and 245 nm) and MSMS spectra as shown in Figure 5. The hypothetical mass fragmentation was exhibited in Figure S3 in the Supplementary Materials.

### 3.4. Colorimetric Analysis

**Figure 6:**
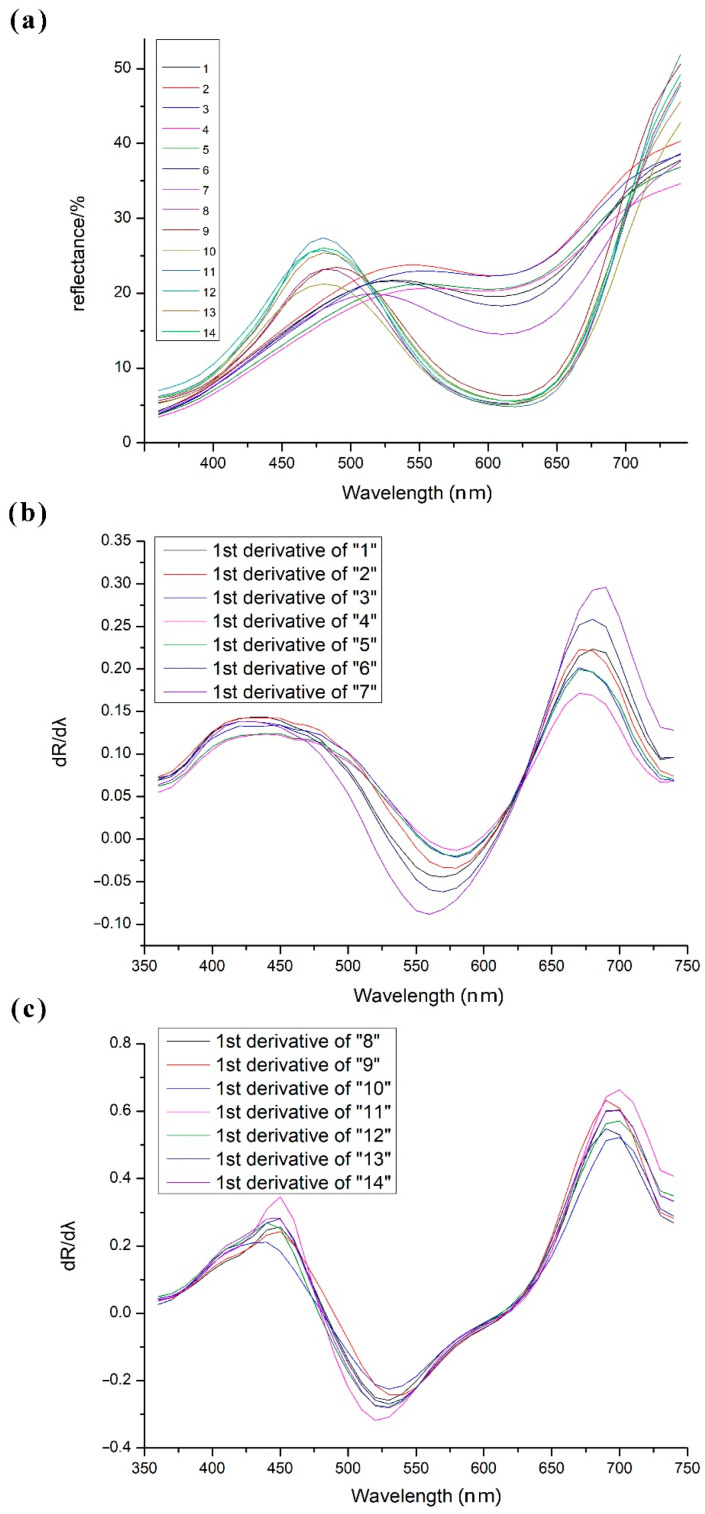
Reflectance spectra of the curtain relics: (**a**) raw spectra of no. 1–14; first derivative curves of (**b**) no. 1–7 and (**c**) no. 8–14 (no. represents the marked numbers).

UV–Vis diffuse reflectance spectra, which were used to measure the color differentiation, indicated two trends corresponding to the faded (no. 1–7) and unfaded (no. 8–14) parts, respectively, where the numbers represent different micro-analysis positions, as shown in Figure 1b. The characteristic positions in the spectral region for the faded and unfaded groups were at around 520 nm and 470 nm, respectively (Figure 6a), and the colorimetric coordinates for fourteen different positions had been reported in Table S1. The curve changes in the fading process revealed the composition and color transformation. There were also some subtle distinctions for the faded ones. The variation tendencies for no. 2 and 3 as well as no. 4 and 5 were basically similar. The color of no. 7 was the darkest, so its curve was in the lowest location. The first derivatives (dR/dλ) could provide more information about the change in reflectance with the wavelength as well as the position of the maximum absorption peak. The results showed a good trend of the increased reflectivity from the faded parts to the unfaded ones, in addition to matching of the main absorption ca. at 440 nm and 670 nm of the former parts (Figure 6b) as well as 450 nm and 700 nm of the latter ones (Figure 6c), respectively. Due to the whole degradation process of the triphenylmethane dye, the weakened resonance in the organic conjugated structures resulted in decreased electron delocalization, and thus, the decolorization produced the hypsochromic shift. The conditions were relatively homogeneous, since the max absorbance peak positions were almost unchanged after the first derivation of the two curve groups, with nuanced differences arising from microadhesives.

### 3.5. Kinetics Investigation of the Photoageing Process

**Figure 7:**
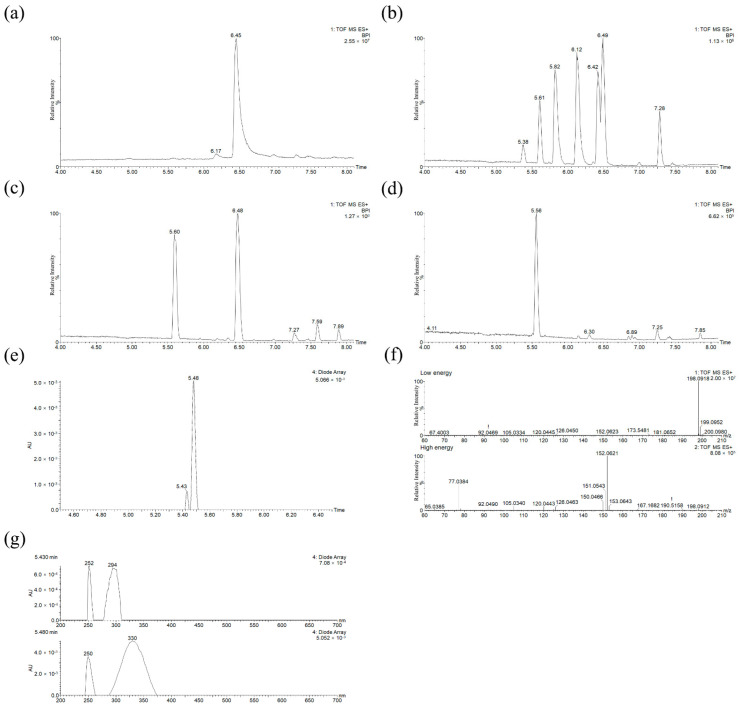
MG representative chromatograms by UPLC-ESI-QTOF MS in a positive ion mode under different aging time: (**a**) K0, 0 h; (**b**) K26, 72 h; (**c**) K27, 78.5 h; (**d**) K28, 96 h; (**e**) co-elute liquid chromatogram; (**f**) MS spectra with an MS^E^ of low and high energy under an RT of 5.56 min; and (**g**) UV–Vis absorbance spectra under an RT of 5.43 and 5.48 min (offset 0.08).

**Figure 8:**
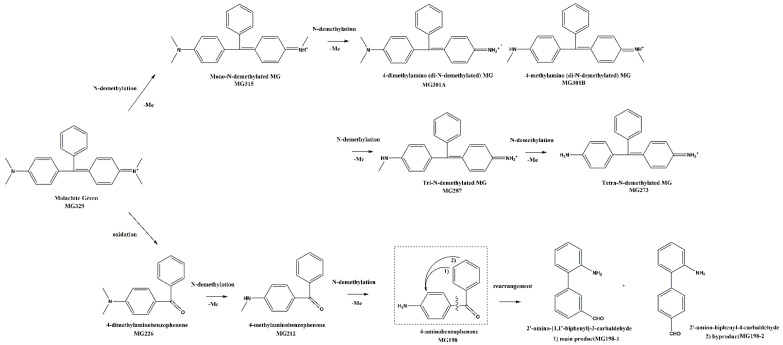
MG degradation pathways under the photo-kinetics simulated aging experiment.

To better understand the aging details, photocatalytic dye degradation was undertaken to estimate the deterioration products. The same LC–MS method was applied for both the kinetic investigation experiment product and the dye extract analysis, and the characteristic results under different aging time of K0, 0 h; K26, 72 h; K27, 78.5 h; and K28, 96 h were exhibited, respectively, in Figure 7. The conjugated chromophore structure of MG molecules was cleaved from K7 with an irradiation time of 10 h. In the whole aging process, there were two main pathways involved: N-demethylation and the cleavage oxidation reactions of central C=C double bonds, displayed in Figure 8. In this study, nine intermediates, as previously reported, were identified, and detailed information is listed in Table S3 in the Supplementary Materials.

In the first pathway during the photoageing process, five species of N-demethylation intermediates, including mono-, di-, tri-, and tetra-N-demethylated compounds, appeared step-by-step. Similar to the reported process [26], the photocatalytic products could also be generated without additional photocatalysts, such as metal oxides or biological enzymes. In the second pathway, the reactions of N-demethylation and oxidation, as well as the molecular rearrangement of pseudomolecular ion *m/z* 198 [M+H]^+^, were referred. The formation of 4-dimethylaminobenzophenone (*m/z* 226) [11] suggested degradation via the oxidation of the central carbon–carbon double bonds of MG followed by two consecutive N-demethylations into 4-methylaminobenzophenone (*m/z* 212) and 4-aminobenzophenone (ABP, pseudo-molecular ion *m/z* 198, [M + H]^+^). Notably, the ABP molecular structure with non-linear optical behavior was not planar subject to its steric hindrance [27]; according to the detection of molecular fragment ion *m/z* 152 (Figure 7f) in the high-energy channel of the MS^E^ mode, the biphenyl coupling product via the electrophilic aromatic substitution (EArS) reaction was concluded. Under certain catalytic conditions in the light chamber, the breaking of the molecular center bond provided rearrangement isomers via the Friedel–Crafts alkylation reaction [28]. NH_2_, as a donating group, favored its electrophilic action of an *ortho* position and formed an aromatic phenyl substitution [29]. Since C=O was an electrowithdrawing substituent, the *meta*-arylated position was the main product [30]. Therefore, *meta*-substitute 2′-amino-[1,1′-biphenyl]-3-carbaldehyde, as the main product, and *para*-position byproduct 2′-amino-biphenyl-4-carbaldehyde both formed with the same pseudo-molecular ion *m/z* 198 in a positive ion mode.

N-demethylation, as the main reaction under the photo-kinetics simulated conditions, occurred in the early aging experiment followed by the second pathway from K26. After irradiation for 78.5 h at K27, the MG dye effectively decomposed. The complete decolorization of the MG solution was found until K28 with an irradiation time of 96 h. After that, no more significant changes could be observed.

### 3.6. Discussion of the Fading Mechanism of MG Industrial Dye in Textile Objects

**Figure 9:**
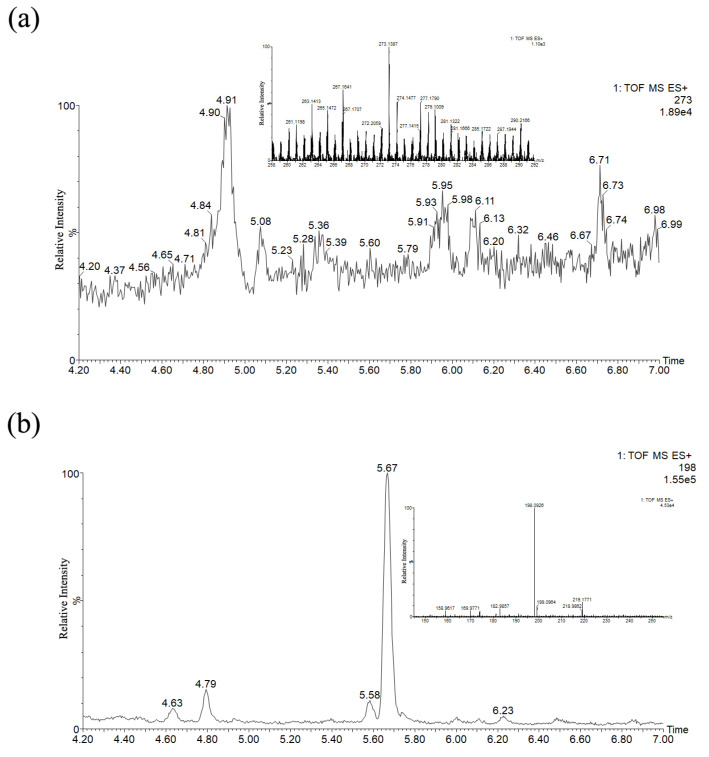
EIC intensities comparison of the first (**a**) and second (**b**) pathways of the final products in the faded curtain.

In the artificial accelerated photoageing experiment, malachite green degraded in methanol under the action of the high reactivity of superoxide species or free radicals. There were many ways to introduce the trace metal ions as photocatalysts that generated reactive oxygen species (ROS) [31], such as the solvent impurities introduced by industrial synthesis [32] and the equipment used or exposed during the aging experiments, which inevitably promoted the catalytic reaction, though a longer aging time was required. Similarly, it was impossible to avoid contact with the metal utensils during the entire dyeing process. These promoted the radicals with the aid of electron–hole pairs, which were excited by the input energy under irradiation [33]. Many trace metal ions, such as Na, Mg, K, Ca, Cu, Zn, Fe, and Mn, could also be introduced at the stage of the formation of silk fibroins [34], and participated in the degradation reaction of MG in a certain humidity environment (ca. 60%RH in this experiment) [35]. The whole fading process involved radical mechanisms in addition to the N-demethylation reaction; that is, under UV-visible irradiation, oxidation reacted with the help of hydroxyl radicals caused by solvent radiation. It is worth noting that there were also the degraded products in the unfaded parts. Shinde et al. reported that cationic MG could also be degraded with photon-free catalysts by donating electrons from the metal core or by creating hydroxyl radicals [31]. Now that this is unavoidable, the faded textiles should strictly be kept away from light in a certain humidity of 50–65% RH, according to a preventive conservation principle from the International Institute for the Conservation of Historic and Artistic Works (IIC) that humidity levels over 65% may cause mold, corrosion, and shrinkage, and that dryness levels under 50% may cause breakage and desiccation. A light exposure limit value should not exceed 50,000 l ×⋅h/year for specific exhibitions. The two pathways mentioned were competitive with each other [33], and both existed in the faded fabrics. From the aspects of the extracted ion chromatograms (Figure 9), where the final product ion signal intensities in the second pathway were 10 times those of the first one, the second pathway was the main reaction, which conformed to the opinion that the cleavage of the central carbon–carbon double bonds of MG was more feasible under acidic conditions [26], which were caused by the photodeterioration of fibers in addition to the yellowing [36].

### 3.7. Metabolomics Analysis for Small Molecules

**Figure 10:**
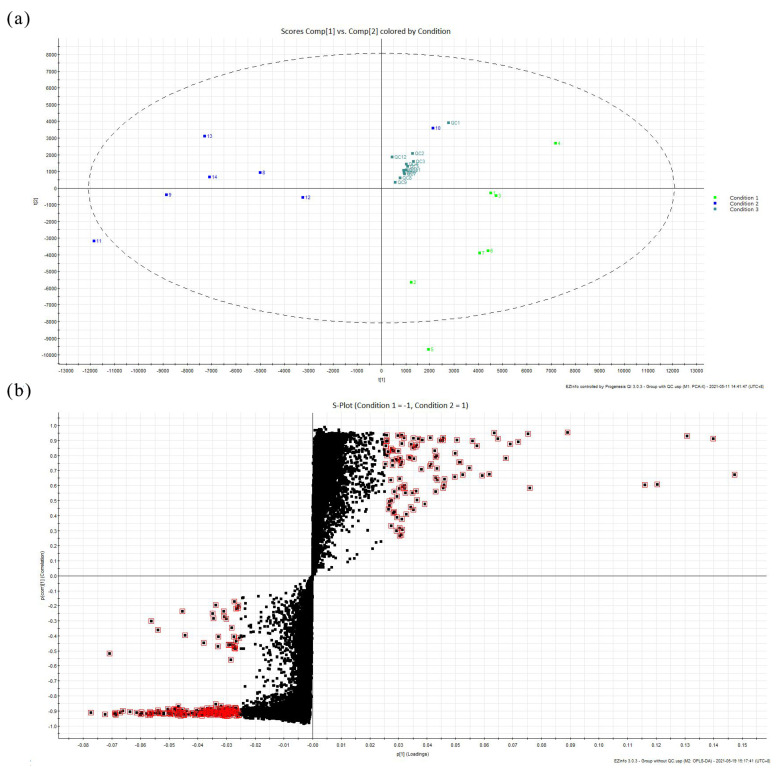
Multivariate analysis of the curtain: (**a**) PCA score plot; (**b**) S-Plot by the OPLS-DA model.

The curtain was applied in partition windows in Qing imperial buildings, and a certain adhesive highlighted with UV fluorescence as reported in Figure S2 was used. These microscopic composition changes were difficult to confirm, and therefore the metabolomic analysis was performed with high sensitivity. To assess all of the runs in the experiment for suitability, QC7 was selected as the reference to describe the chemical information from all 26 injections and then conducted peak picking and alignment reviewing, with all of the scores being above 80, indicating that the individual differences were relatively unremarkable. According to the PCA score plot (Figure 10a), there were three general groups, including Condition 1, presenting seven samples from the faded parts with green squares; Condition 2, presenting seven samples from the unfaded parts with lake blue squares; and Condition 3, presenting all of the samples mixed as QC with cyan squares.

A certain difference could be found between no. 10, with visible dirt, and the other unfaded areas. A series of ion peaks with molecular weight differences of 44 belonged to a -CH_2_CH_2_O- ethoxylate unit, meanwhile fragment ions *m/z* 133.08, 177.11, 221.13, 265.16, and 309.18 in the high-energy channel could be detected; consequently, polydisperse poly(ethylene glycol) (pPEG) ((C_2_H_4_O)_n_H_2_O, n was from 8 to 25) was confirmed in the application as a textile cleaning auxiliary [37,38]. Herein, pPEG (n ≥ 17) was found unstable and degraded by losing the -CH_2_O- subunit with fragment ion *m/z* 30 in a positive mode.

A supervised OPLS-DA model, without the participation of QC samples, by compulsorily grouping the faded and unfaded samples was conducted, and the S-Plot is shown in Figure 10b. The obvious markers (VIP > 1) with red squares could analyze the differences and identify the unique components by measuring both the change magnitude and the comparison significance away from the X and Y axes, respectively. These extractions contributed most significantly to the differences between the two groups. The identified results, including the formula, RT (min), isotope similarity, ANOVA (p), q-value, max fold change, and the min CV%, are listed in Table S4, and the relevant molecular structures are exhibited in Figure S4 in the Supplementary Materials. The lysophosphatidylcholine (16:0 and 18:0) metabolites are a typical class of polar lipids and could form an amylose–lipid complex during heating, which was traditionally involved in preparing starch paste [39]. Platelet-activating factor (PAF), a potent phospholipid activator, was also detected [40]. Generally, amylose was one of the polymers in the starch-based system, and the monomer was released and could be recognized when acid hydrolysis occurred, such as in the deterioration of silk or the introduction caused by pretreatment as well as liquid eluent acidity. The identified unsaturated fatty acids, as drying oil components, were often found as binders in the wooden elements of ancient Chinese buildings.

## 4. Conclusions

The purpose of this research was a comprehensive study of the fading mechanism of malachite green (MG) industrial dye, based on an analysis of the chemical composition of a fragment of a marquisette curtain and a simulation study of the identified dye degradation. The fabric weave type was identified as leno jacquard, and the fiber used has been identified as mulberry silk. The results of the colorimetric measurements provided spectral data with two types of curves that represented fragments of the fabric that faded due to exposure to light in addition to fragments that were covered with wooden slats, and thus were unfaded. Malachite green was identified in the samples extracted from the object by means of LC–MS, and a simulation study on MG degradation was conducted through a photo-kinetics aging experiment. Two degradation reactions occurred competitively in the whole aging process, including N-demethylation and oxidation into benzophenone derivatives. The cleavage reaction of the central C=C double bonds was more favorable under the acidity caused by the photodeterioration of the fibers; therefore, the second pathway was more relevant to faded fabric dyed with MG. A chemometric assay was adopted to analyze the differential metabolites using UPLC-ESI-QTOF MS. A statistical analysis that included PCA and OPLS-DA models showed polar lipids and drying oils as different metabolites in the unfaded borders compared to the faded parts; therefore, we draw the conclusion that the adhesive mainly used starch and oil. Furthermore, the presence of pPEG, which was probably used as a cleaning agent, was confirmed in the unfaded parts, covered with dirt. Several similarly faded historic textiles that are collected in the Palace Museum were dyed with industrial dyes in the late Qing Dynasty, and should be highly regarded during their storage and conservation, since the aging and fading of these organic materials are irreversible.

## Data Availability

Not applicable.

## Supplementary Materials

The following supporting information can be downloaded at: https://www.mdpi.com/article/10.3390/molecules27144411/s1. Figure S1: Characteristic color changes in reference MG solution under different aging time: (a) K0, 0 h; (b) K26, 72 h; (c) K27, 78.5 h; and (d) K28, 96 h; Figure S2: The marquisette curtain captured under visible light (a) and UV-induced fluorescence (b), respectively; Figure S3: The hypothetical MSMS fragmentations of MG in a positive ion mode; Figure S4: The identified molecular structures of differential metabolites with an omics analysis in the OPLS-DA model; Table S1: Colorimetric coordinates of fourteen different positions from the marquisette curtain relics; Table S2: Different aging time of simulated malachite green (MG, no. 0–30) in a methanol solvent; Table S3: Detailed information on intermediates identified by LC–MS in the kinetics investigation of the photoageing process in a positive ion mode (K19 contained all of the discovered products as references); Table S4: The identified small molecule metabolites by the OPLS-DA model (both precursor and theoretical fragment tolerance of 5 ppm).
