# Supplementary material for: An Integrated Study on the Fading Mechanism of Malachite Green Industrial Dye for the Marquisette Curtain in the Studio of Cleansing Fragrance, the Palace Museum (Beijing)
Source: Molecules. 2022 Jul 9;27(14):4411. doi: 10.3390/molecules27144411 (PMC9322362; doi:10.3390/molecules27144411)
Supplement: Supplementary file 1 [file molecules-27-04411-s001.zip › molecules-1797288-supplementary.pdf]

Supplementary Materials

An Integrated Study on the Fading Mechanism of Malachite Green Industrial Dye for the Marquisette Curtain in the Studio of Cleansing Fragrance, the Palace Museum (Beijing)

Number of Figures: 4

Number of Tables: 4

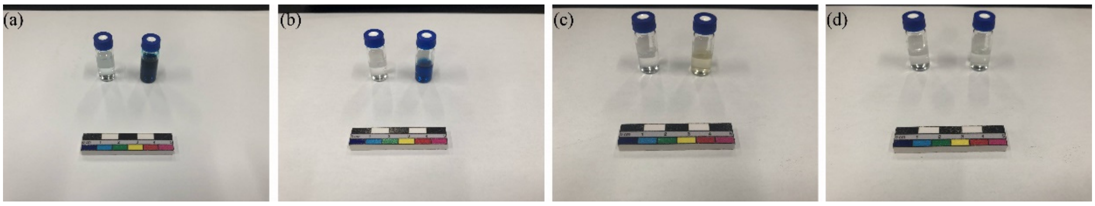

Figure S1. Characteristic color changes of reference MG solution under different ageing time: (**a**) K0, 0 h; (**b**) K26, 72 h; (**c**) K27, 78.5 h; and (**d**) K28, 96 h.

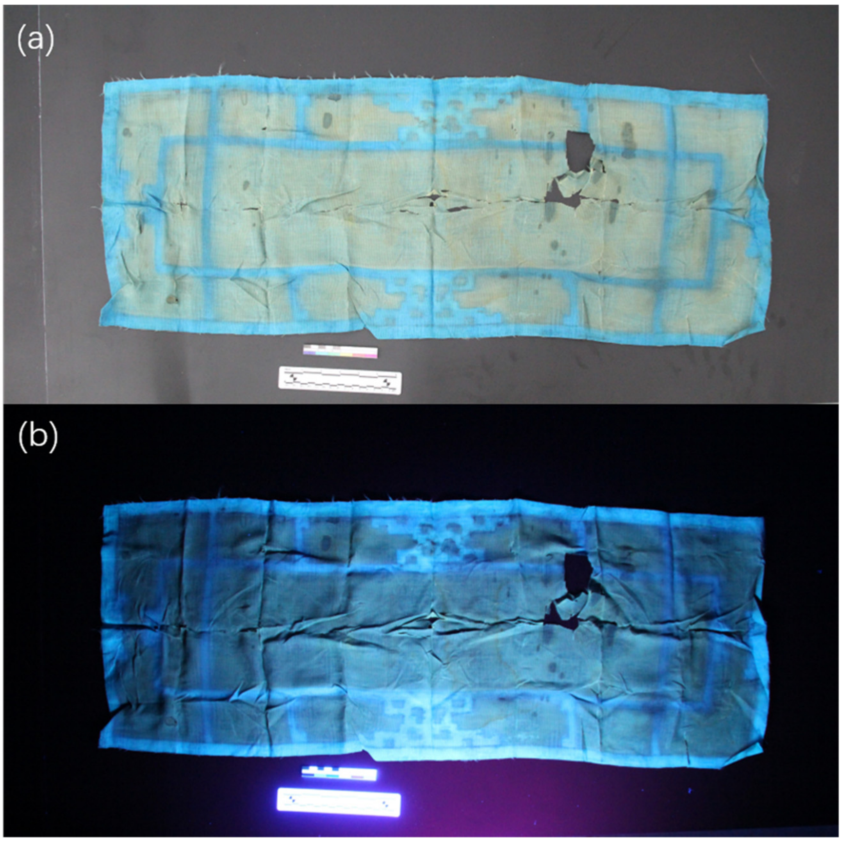

Figure S2. The marquisette curtain captured under visible light (**a**) and UV-induced fluorescence (**b**), respectively.

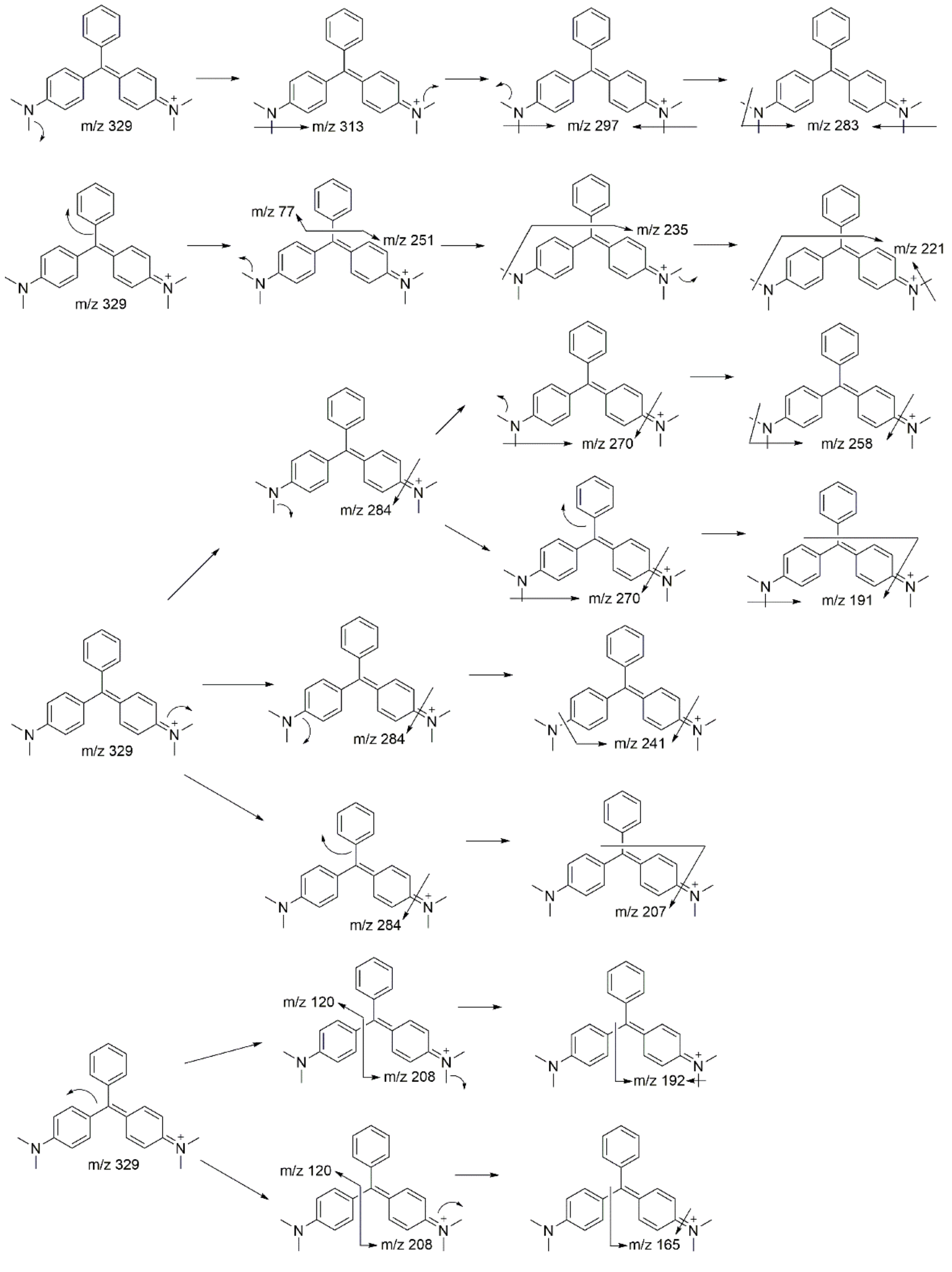

Figure S3. The hypothetical MSMS fragmentations of MG in a positive ion mode.

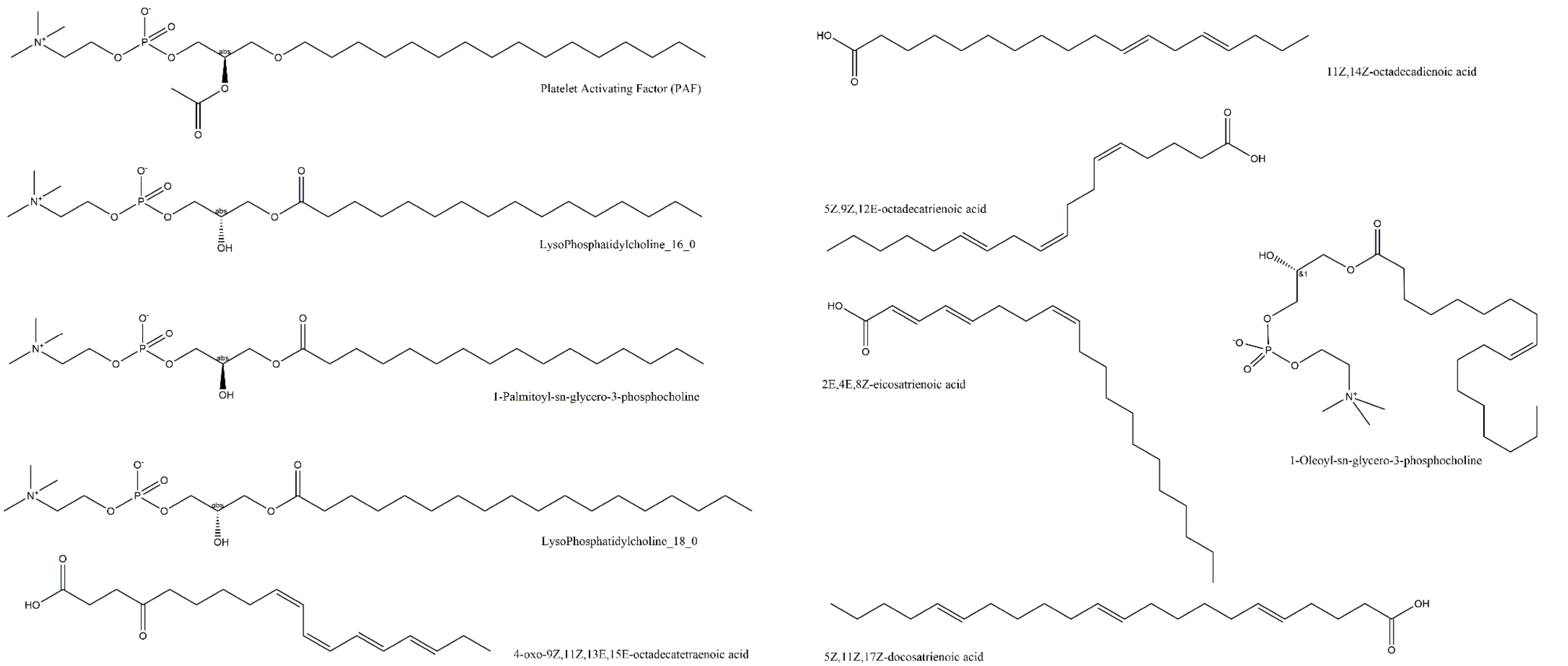

Figure S4. The identified molecular structures of differential metabolites with an omics analysis in the OPLS-DA model.

Table S1. Colorimetric coordinates of fourteen different positions from the marquisette curtain relics.

**Samples**	L^*	a^*	b^*
No. 1	52.38	-5.75	12.38
No. 2	54.64	-4.57	14.76
No. 3	53.91	-3.43	15.93
No. 4	51.52	-2.92	15.81
No. 5	52.08	-3.38	15.36
No. 6	51.85	-6.84	10.91
No. 7	48.80	-9.33	6.80
No. 8	41.38	-20.25	-14.13
No. 9	43.78	-21.19	-9.61
No. 10	40.31	-17.83	-15.03
No. 11	42.41	-22.03	-17.99
No. 12	42.55	-19.29	-18.20
No. 13	43.36	-21.14	-14.47
No. 14	43.32	-20.62	-15.88

Table S2. Different ageing time of simulated malachite green (MG, no. 0–30) in a methanol solvent.

Simulated samples	Ageing time/h	Irradiation/KJ·m^{-2}
K0	0.0	0
K1	1.0	1980
K2	2.0	3960
K3	4.0	7920
K4	6.0	11880
K5	7.0	13860
K6	8.0	15840
K7	10.0	19800
K8	12.0	23760
K9	14.0	27720
K10	15.0	29700
K11	18.0	35640
K12	20.0	39600
K13	22.0	43560
K14	23.0	45540
K15	24.0	47520
K16	30.0	59400
K17	36.0	71280
K18	40.0	79200
K19	46.0	91080
K20	48.0	95040
K21	50.0	99000
K22	56.0	110880
K23	60.0	118800
K24	66.0	130680
K25	70.0	138600
K26	72.0	142560
K27	78.5	155430
K28	96.0	190080
K29	100.0	198000
K30	102.0	201960

Table S3. Detailed information of intermediates identified by LC–MS in the kinetics investigation of the photoageing process in a positive ion mode (K19 contained all of the discovered products as references).

Component name	Abbr.	Adducts	Formula	Observed pseudo-molecular ion (m/z)	Mass error (ppm)	RT/min	Fragment ion (m/z)
Malachite Green	MG329	M+	$C_{23}H_{25}N_2^+$	329.2024	1.8	6.38	313/284/208/165
Mono-N-demethylated MG	MG315	M+	$C_{22}H_{23}N_2^+$	315.1855	-0.3	6.12	299/270/194/165
4-dimethylamino (di-N-demethylated) MG	MG301A	M+	$C_{21}H_{21}N_2^+$	301.1698	-0.6	5.66	285/256/180/165
4-methylamino (di-N-demethylated) MG	MG301B	M+	$C_{21}H_{21}N_2^+$	301.1696	-1.0	5.82	285/270/194/106
Tri-N-demethylated MG	MG287	M+	$C_{20}H19N_2^+$	287.1543	0.1	5.37	271/254/180/165
Tetra-N-demethylated MG	MG273	M+	$C_{19}H_{17}N_2^+$	273.1382	-1.4	4.97	256/180
4-dimethylaminobenzophenone	MG226	M+H	$C_{15}H_{15}NO$	226.1225	-0.4	7.28	210/134/106/77
4-methylaminobenzophenone	MG212	M+H	$C_{14}H_{13}NO$	212.1068	-0.9	6.50	196/120/105/77
2'-amino-[1,1'-biphenyl]-3-carbaldehyde	MG198-1	M+H	$C_{13}H_{11}NO$	198.0918	-0.5	5.48	152/120/92/77
2'-amino-biphenyl-4-carbaldehyde	MG198-2	M+H	$C_{13}H_{11}NO$	198.0918	-0.5	5.43	152/120/92/77

Table S4. The identified small molecule metabolites by the OPLS-DA model (both precursor and theoretical fragment tolerance of 5 ppm).

Compound	Adducts	Formula	RT	m/z	Score	Fragmentation score	Mass error (ppm)	Isotope similarity	ANOVA (p)	q Value	Max Fold Change	Highest mean	Loweast mean	Min CV %
Platelet-activating factor (PAF)	M+H, 2M+H, M+Na	$C_{26}H_{54}NO_7P$	9.69	524.3714	41.6	14.2	0.56	94.45	8.66E-06	2.16E-06	13.98	Condition 2	Condition 1	25.18
lysophosphatidylcholine_16_0	M+H	$C_{24}H_{50}NO_7P$	8.45	496.3403	47.8	43	1.00	97.30	0.00747	0.00496	78.5	Condition 2	Condition 1	29.94
1-Oleoyl-sn-glycero-3-phosphocholine	M+H, 2M+H, M+Na	$C_{26}H_{52}NO_7P$	8.24	521.3495	43.8	31.1	2.61	90.95	1.10E-06	3.86E-07	107.47	Condition 2	Condition 1	24.69
1-Palmitoyl-sn-glycero-3-phosphocholine	M+H	$C_{24}H_{50}NO_7P$	8.65	496.3403	47.8	43	1.00	97.30	0.00747	0.00496	78.5	Condition 2	Condition 1	29.94
PC(18:0/0:0)	M+H, 2M+H, M+Na	$C_{26}H_{54}NO_7P$	8.87	524.3714	39.6	4.26	0.56	94.45	0.00143	0.00134	14.1	Condition 2	Condition 1	25.18
4-oxo-9Z,11Z,13E,15E-octadecatetraenoic acid	M+H	$C_{18}H_{26}O_3$	7.34	291.1951	39.2	2.07	-1.19	95.43	1.42E-09	2.23E-08	5.82	Condition 2	Condition 1	17.47
11Z,14Z-octadecadienoic acid	M+H	$C_{18}H_{32}O_2$	7.95	281.2474	48.3	45.2	-0.27	96.57	4.43E-07	2.94E-06	2.96	Condition 2	Condition 1	16.88
5Z,9Z,12E-octadecatrienoic acid	M+H	$C_{18}H_{30}O_2$	9.01	279.2317	39.6	1.47	-0.46	96.94	1.38E-06	7.35E-06	3.18	Condition 2	Condition 1	20.95
2E,4E,8Z-eicosatrienoic acid	M+H	$C_{20}H_{34}O_2$	9.74	307.2628	40	6.87	-1.17	94.46	2.52E-07	1.89E-06	2.62	Condition 2	Condition 1	16.34
5Z,11Z,17Z-docosatrienoic acid	M+H	$C_{22}H_{38}O_2$	10.69	335.2945	39.4	1.32	0.08	95.63	1.7E-09	2.62E-08	3.48	Condition 2	Condition 1	8.55
